# Justified Suspicion: Symptomatic Syphilitic Alopecia in a Patient with Well-Controlled HIV

**DOI:** 10.1155/2021/1124033

**Published:** 2021-11-15

**Authors:** Robert Jame, Yousif Al-Saeigh, Leo L. Wang, Kevin Wang

**Affiliations:** ^1^Sackler School of Medicine, 17 E 62nd St, New York, NY 10065, USA; ^2^Pennsylvania Hospital, University of Pennsylvania Health System, Philadelphia, PA, USA; ^3^Robert Wood Johnson Medical School, 125 Paterson St, New Brunswick, NJ 08901, USA

## Abstract

**Background:**

An estimated 25% of primary and secondary syphilis, a sexually transmitted infection caused by the spirochete bacterium *Treponema pallidum*, occurs in patients coinfected with human immunodeficiency virus (HIV) (Chesson et al., 2005). This association is especially evident in men who have sex with men (MSM). In HIV-positive patients, primary syphilis infection may progress more rapidly to the tertiary, and most destructive, stage and reinfection can start with the latent or tertiary stage; in such patients, advanced syphilis may arise without clinical warning signs (Kenyan et al., 2018). It is important to note that neurosyphilis can occur during any stage of infection in all patients, regardless of immunocompetence status (CDC, 2021). Case Presentation. A 56-year-old male with a past medical history of well-controlled HIV with a CD4 count of 700 cells/mm^3^ and an undetectable viral load, psoriasis, and a remote episode of treated syphilis, presented with a two-week history of a diffuse desquamating rash, alopecia, sinusitis, unilateral conjunctivitis, and blurred vision. His last sexual encounter was over ten months ago. The diagnosis of syphilis was confirmed by microhemagglutination assay, and he was treated for presumed neuro-ocular infection with a two-week course of intravenous Penicillin G.

**Conclusion:**

Syphilis has acquired a reputation as “the great masquerader” due to its protean manifestations. It may follow an unpredictable course, especially in HIV-positive patients, including those whose treatment has achieved undetectable serology. For example, ocular syphilis may present in an otherwise asymptomatic individual (Rein, 2020) and alopecia may arise as the sole indication of acute syphilitic infection (Doche et al., 2017). Therefore, a high index of suspicion is warranted in order to prevent severe and irreversible complications.

## 1. Introduction

Although it remains relatively rare, the incidence of syphilis has been rising in the US after decreasing by 90% from 1990 to 2000 [1]. In 2018, 35,063 cases of primary and secondary (P&S) syphilis were reported, the highest number since 1993. MSM accounted for 53.5% of these. Of said MSM whose HIV status was known, 41.6% were coinfected with the virus [2]. Suggested causes include an increase in risky sexual behavior (i.e., risk compensation) among those taking preexposure prophylaxis (PrEP) [3].

The rate of P&S syphilis also increased among women by 172.7% during 2014–2018. An increasing incidence of syphilis has been noted in all geographic states and in all racial and ethnic groups [4]. It has been suggested that the use of dating apps has played a role in the increased incidence of syphilis in both MSM and other genders and behavioral categories [5].

Reported cases of ocular syphilis have also increased. Clusters of ocular syphilis in Seattle, Washington, and San Francisco, California, were reported in 2015, prompting the CDC to issue a clinical advisory [6–8]. Ocular syphilis is a form of neurosyphilis which may occur with or without CNS involvement. Posterior uveitis and panuveitis are its most common presentations, although it may affect nearly any structure of the eye. Other manifestations include anterior uveitis, interstitial keratitis, retinal vasculitis, retinitis, chorioretinitis, and optic neuropathy. A case of ocular syphilis is defined by the CDC as a patient with syphilis of any stage and clinical symptoms or signs consistent with ocular disease [8,9].

## 2. Case Report

Our patient was a 56-year-old man diagnosed with HIV in 1991. He reported compliance with antiretroviral therapy (abacavir-dolutegravir-lamivudine, 1 tablet PO QD, and tenofovir, 300 mg PO QD) with a CD4 count of 700 cells/mm^3^ and an undetectable viral load. He identified as an MSM and lived with a male partner. He presented to our emergency department with a chief complaint of sinus headaches and rash.

His constellation of symptoms began with an unintentional weight loss of about 15 pounds over the previous 8 months. Several weeks prior, he developed nonpruritic and nonpainful erythematous, scaly plaques and alopecia. The plaques first appeared on his palms and soles and then spread to his arms, shins, groin, abdomen, and scalp. He denied recent travel or outdoor activities such as would have been consistent with rickettsial disease. The patient endorsed generalized hair loss, including his scalp and especially his eyebrows. Over the past week, he had also developed loss of appetite and fatigue.

The rash co-occurred with sinus headaches associated with nasal congestion and left eye redness, discharge, and pain. The ocular pain alternated between dull and stabbing. On questioning, he endorsed left eye floaters. He noticed a left eye visual deficit only after his right eye was covered during examination. His partner also developed unilateral eye redness, decreased vision, and a rash over his thorax two months ago, for which he was diagnosed with herpes simplex and treated with famciclovir and a topical steroid. Our patient denied fevers or chills at home, arthralgias, neck stiffness, and focal neurologic deficits. His last sexual encounter was over ten months ago, not with his partner but with a friend. He used marijuana recreationally, but no other illicit drugs. He smoked cigarettes daily.

His past medical history included a diagnosis of syphilis 10–12 years ago for which he was treated.

On arrival, his vital signs were as follows: blood pressure, 120/76 mmHg; pulse, 86 bpm; oral temperature, 99.3°F (37.4°C); SpO_2_, 97% on room air; respiratory rate, 18 bpm; weight, 154.8 lbs (70.2 kg); and BMI, 19.88 kg/m^2^. Physical examination revealed decreased vision and scleral injection of the left eye as well as diffuse scaling and desquamating erythematous plaques over his palms and soles (Figures 2 and 3). Over his extremities and trunk were found erythematous macules and plaques with overlying thin, white scaling rings consistent in appearance with Biett collarettes.

Laboratory workup showed strongly positive RPR at 1 : 256. A positive MHA-TP (Microhemagglutination Assay for Treponemal palladium Antibodies) confirmed the diagnosis of syphilis. His CD4 count during admission was measured at 250 cells/mm^3^ with 21% CD4 lymphocytes and his viral load at 220,000 copies/mL. As our patient reported compliance with his antiretroviral regimen, HIV genotyping studies were ordered to assess for resistance.

Our patient was treated with a two-week course of intravenous Penicillin Q4H and monitored during the first 48 hours of treatment for Jarisch–Herxheimer reaction. He was also diagnosed with subacute anterior and intermediate uveitis of the left eye and prescribed prednisolone drops Q2H while awake, cyclopentolate QD, and ofloxacin BID (in case of bacterial conjunctivitis), each to be administered to his left eye. In addition, he was prescribed prednisolone drops QID for his right eye. Follow-up was scheduled with ophthalmology and, regarding his HIV status, infectious disease. At this three-month follow-up with ophthalmology and infectious disease, all his symptoms resolved, including his alopecia and rash.

## 3. Discussion

A Biett collarette, or Biett's sign, is defined by a ring of scales encircling or contained within a circumscribed lesion (Figure 3). It is a hallmark of secondary syphilis which may differentiate “the great masquerader” from other conditions that manifest annular maculopapular morphology and scaling. Biett collarettes classically present on the palms and soles but, in our patient, were more visible on the extremities and trunk (those on his palms and soles may have been overtaken by excoriation).

The alopecia affecting our patient's eyebrows and scalp was consistent in appearance with the “moth-eaten” variant (Figure 1) of alopecia syphilitica (AS), which is relatively specific to secondary syphilis. The reported incidence of AS in secondary syphilis ranges from 4 to 12.5 percent [10–13]. AS is a noninflammatory, nonscarring type of alopecia divided into two types: symptomatic, which is more common and co-occurs with other mucocutaneous changes, and essential, in which alopecia is the only skin finding [14]. Loss of hair in AS may develop according to three patterns: moth-eaten, diffuse, or a combination thereof called mixed. The moth-eaten appearance is the most common of these and may be mistaken for other types of alopecia (e.g., alopecia areata, tinea capitis, and trichotillomania), as the clinician should be aware, especially because AS may be the only presenting feature of syphilitic infection [13,15–17]. Syphilitic alopecia is also associated with cerebrospinal syphilis [17]. Alopecia from syphilis is expected to resolve within several months of treatment.

AS may be differentiated by histopathology and trichoscopy; however, it is important to note that biopsy may also be indistinguishable from other types of alopecia: serology and other symptoms consistent with syphilis are its distinguishing findings [18]. In a histopathological comparison of nine patients with AS versus 13 cases of alopecia areata, Lee and Hsu found that the latter is characterized by peribulbar eosinophils. It was also found that, in the absence of peribulbar eosinophils, AS is suggested by parabulbar lymphoid aggregates, an abundance of lymphocytes in the isthmus, or the presence of plasma cells [19]. The punch biopsy taken from our patient, significant for diffuse inflammatory infiltrate with plasma cells, was consistent with these findings. Jordaan and Louw found that follicular plugging, sparse perivascular and perifollicular lymphocytic infiltrate, telogenization, and follicle-oriented melanin clumping are specific to the moth-eaten pattern [19].

Reported trichoscopic findings associated with moth-eaten AS include zigzag hairs, [20] yellow dots at the center of alopecia patches and black dots at their periphery, [21] black dots at any location within lesions, irregularly dilated capillaries with small blood extravasation, [22] focal atrichia, hypopigmented hair shafts, [21] reduced number of terminal hairs, empty hair follicles, vellus hairs, and red-brown background [22]. In patients with mixed-pattern AS, Tognetti et al. reported a number of these findings (including reduced number of terminal hairs, yellow and black dots, erythematous background, dilated capillaries, and vellus hair) in addition to the new findings of “tapered bended hairs” (single- or double-bending tapering hairs), focal follicular hyperkeratosis, and diffuse scaling [23]. Consistent with these findings, our patient presented with diffuse scaling as well.

Our differential included secondary syphilis, psoriasis, Stevens–Johnson syndrome, vasculitis, scabies, tick-borne illness, medication reaction, and malignancy (i.e., cutaneous T-cell lymphoma). Although syphilis offered a possible explanation for our patient's low CD4 count, his viral load was erratically high; this underscored the necessity of testing for antiretroviral resistance. On admission, our patient had a normal white blood cell count of 7.1 K/*µ*L, an elevated C-reactive protein level of 23.9 mg/L, and an elevated erythrocyte sedimentation rate of 62 mm/hr. Chest X-ray was unremarkable. Cultures were negative for trichomonas and chlamydia. Antinuclear antibody and antineutrophil cytoplasmic antibody assays were negative. CT imaging of the orbits was unremarkable.

Neurosyphilis, including that with ocular involvement, can present during any stage of infection [24]. For this reason, a revised case definition for syphilis was introduced by the CDC in 2018. Changes include replacing the term “early latent syphilis” with “syphilis, early non-primary non-secondary” to reflect the fact that neurologic symptoms can occur during this stage [2, 25]. In a literature review of 101 patients with HIV and ocular syphilis, 21 (or 39%) of the 54 patients with clinical manifestations presented with visual symptoms only [26].

Ocular syphilis is a form of neurosyphilis which may occur with or without CNS involvement. Posterior uveitis and panuveitis are its most common presentations, although it may affect nearly any structure of the eye. A nonexhaustive list of additional associations includes anterior uveitis, interstitial keratitis, retinal vasculitis, retinitis, chorioretinitis, and optic neuropathy. A case of ocular syphilis is defined by the CDC as a patient with clinical symptoms or signs consistent with ocular disease with syphilis of any stage [8,9]. Ocular syphilis can occur in immunocompetent patients without underlying disease [10] and in HIV+ patients receiving highly active antiretroviral therapy with normal CD4 cell counts [27, 28]. Having over 28 days of ocular symptoms before diagnosis is associated with a poor prognosis [29]. A retrospective chart review (1984–2014) of patients with ocular syphilis conducted at the Johns Hopkins Hospital in Baltimore, Maryland, revealed 7% and 6% incidence rates of legal blindness in HIV- and HIV+ patients, respectively, in cases/eye-years (i.e., cases/number of eyes *∗* number of years followed) [9].

All syphilitic infection with ocular involvement must be treated for presumed neurosyphilis, regardless of cerebrospinal fluid analysis findings [30]. Corticosteroids are commonly used to treat inflammatory eye conditions. However, if not coadministered with antibiotic therapy, steroids can increase the treponemal load of ocular syphilis patients, possibly leading to permanent vision loss and other effects of disease progression [31–33]. For this reason among others, including its variable presentations and devastating potential, the physician should not hesitate to include syphilis in the differential diagnosis of those with uveitis, vision loss, alopecia, or other suggestive findings and should complete a confirmatory assessment.

## 4. Conclusion

Syphilis can present with characteristic findings or in a far more idiosyncratic manner. It is also known for mimicking other pathologies. In susceptible patients, advanced syphilis may present earlier than previously expected. Because its incidence is rising, syphilis requires a high degree of suspicion.

## Data Availability

No data were used to support this study.

## Supplementary Materials

Supplementary Materials
*Figure 1.* “Moth-eaten” alopecia syphilitica, as seen in this patient's eyebrows, is a rare, but highly indicative, finding of secondary syphilis. *Figure 2.* The rash began with diffuse scaling and desquamating erythematous plaques over his palms and soles, which spread centripetally. *Figure 3.* Biett collarettes, a hallmark of secondary syphilis, are defined by a ring of scales encircling or contained within a circumscribed lesion. Classically, they present on the palms and soles. One is visible on our patient's right sole (indicated by the arrow). More may have been present but overtaken by excoriation. They were found in greater numbers over his trunk and extremities.
