# Supplementary figures and images for: Justified Suspicion: Symptomatic Syphilitic Alopecia in a Patient with Well-Controlled HIV
Source: Case Rep Infect Dis. 2021 Nov 15;2021:1124033. doi: 10.1155/2021/1124033 (PMC8608535; doi:10.1155/2021/1124033)

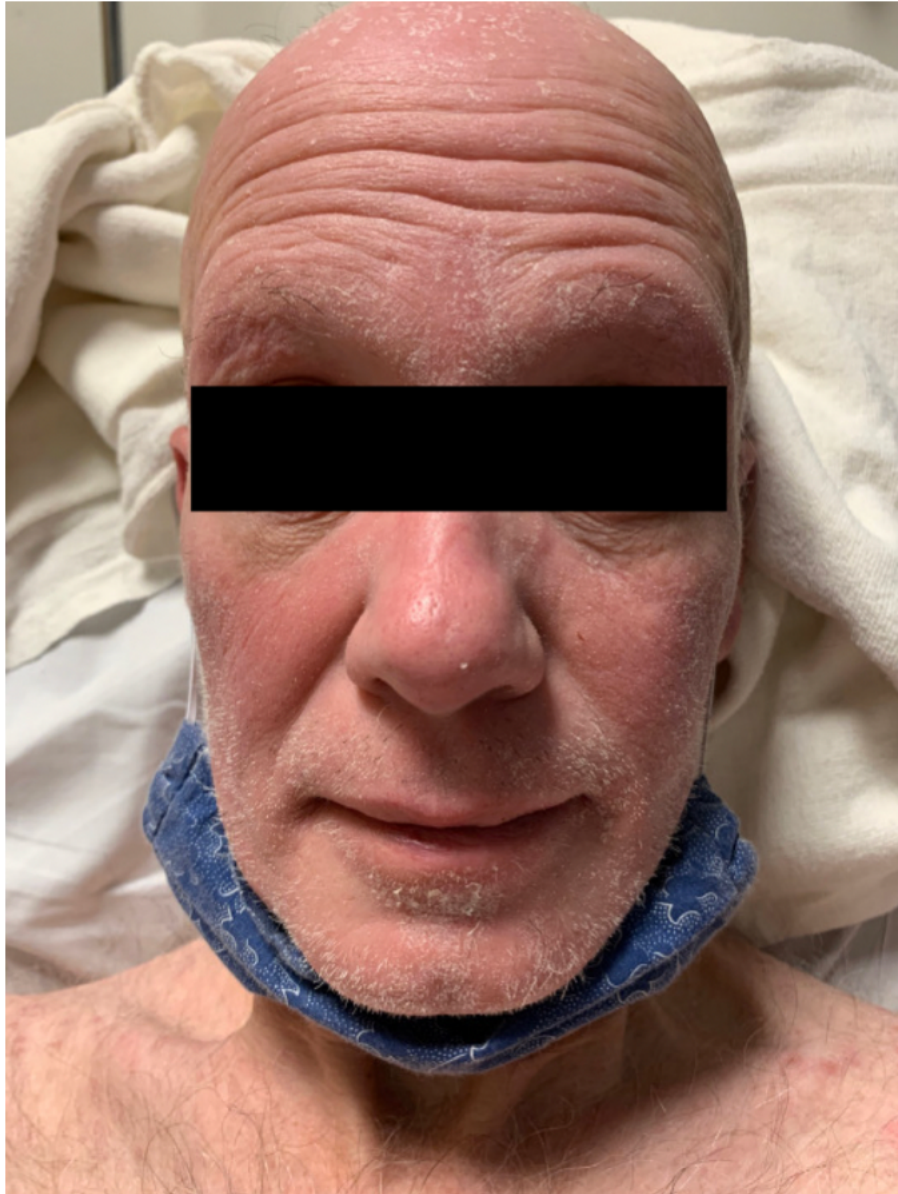

Supplement: Supplementary Materials — Figure 1. “Moth-eaten” alopecia syphilitica, as seen in this patient's eyebrows, is a rare, but highly indicative, finding of secondary syphilis. Figure 2. The rash began with diffuse scaling and desquamating erythematous plaques over his palms and soles, which spread centripetally. Figure 3. Biett collarettes, a hallmark of secondary syphilis, are defined by a ring of scales encircling or contained within a circumscribed lesion. Classically, they present on the palms and soles. One is visible on our patient's right sole (indicated by the arrow). More may have been present but overtaken by excoriation. They were found in greater numbers over his trunk and extremities. [file 1124033.f1.zip › 1124033.f1/Figure1 (1).pdf]

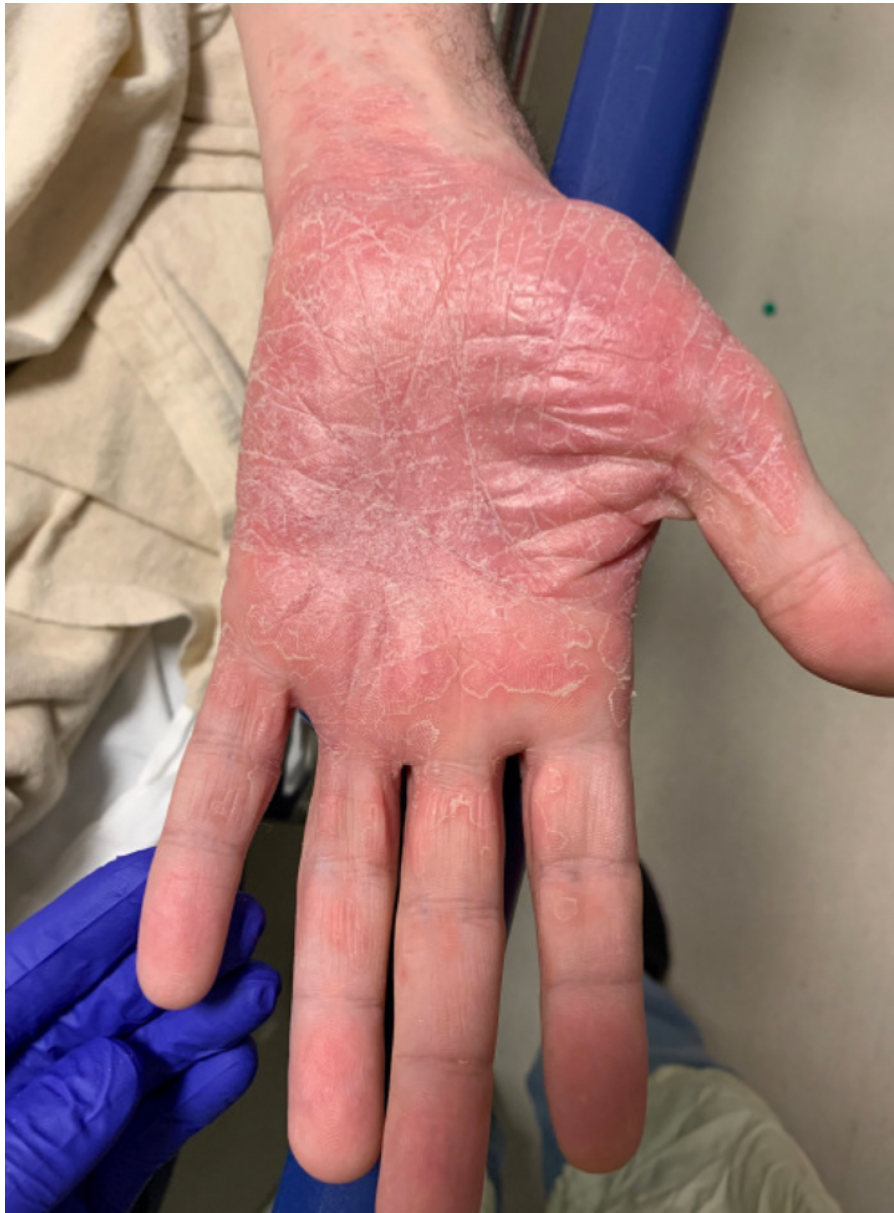

Supplement: Supplementary Materials — Figure 1. “Moth-eaten” alopecia syphilitica, as seen in this patient's eyebrows, is a rare, but highly indicative, finding of secondary syphilis. Figure 2. The rash began with diffuse scaling and desquamating erythematous plaques over his palms and soles, which spread centripetally. Figure 3. Biett collarettes, a hallmark of secondary syphilis, are defined by a ring of scales encircling or contained within a circumscribed lesion. Classically, they present on the palms and soles. One is visible on our patient's right sole (indicated by the arrow). More may have been present but overtaken by excoriation. They were found in greater numbers over his trunk and extremities. [file 1124033.f1.zip › 1124033.f1/Figure2 (1).pdf]

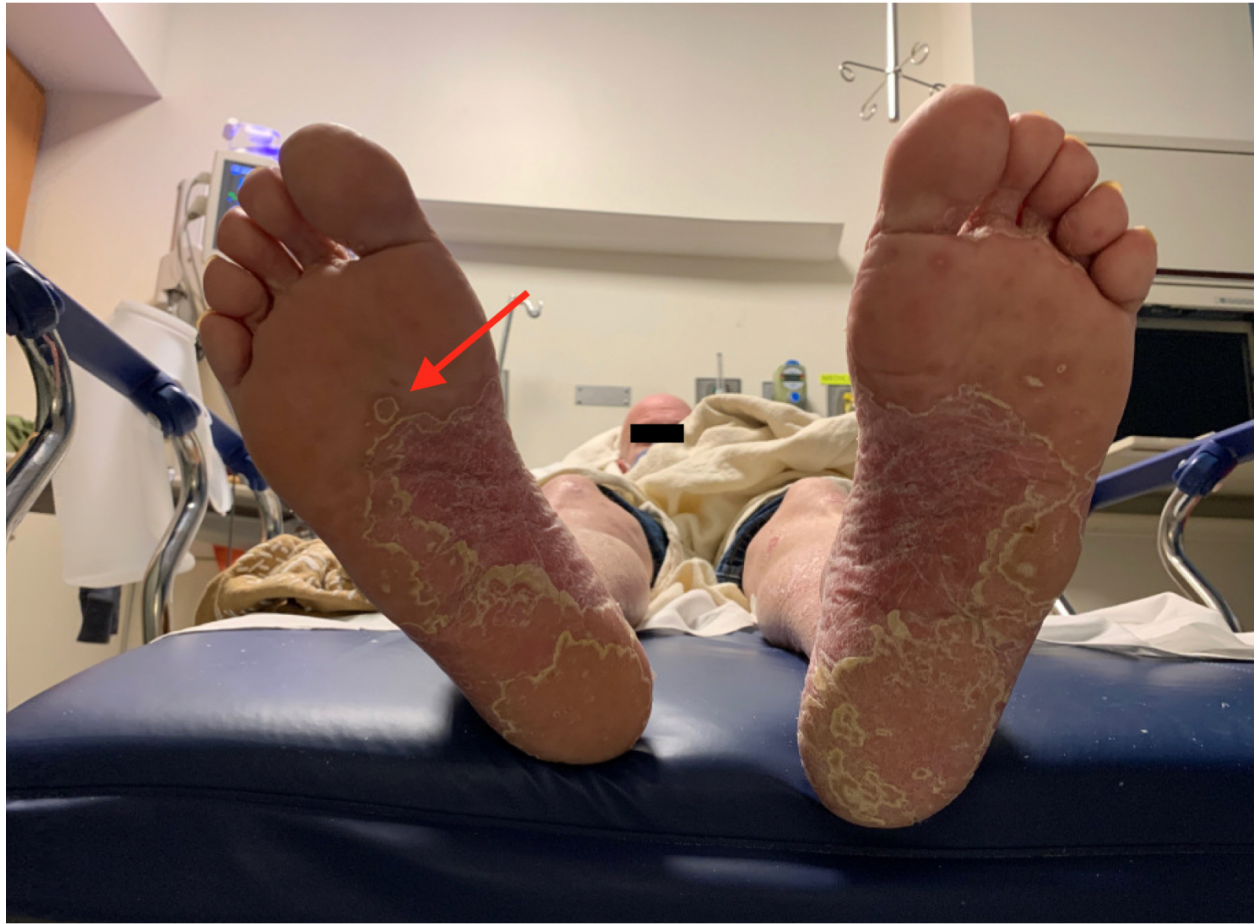

Supplement: Supplementary Materials — Figure 1. “Moth-eaten” alopecia syphilitica, as seen in this patient's eyebrows, is a rare, but highly indicative, finding of secondary syphilis. Figure 2. The rash began with diffuse scaling and desquamating erythematous plaques over his palms and soles, which spread centripetally. Figure 3. Biett collarettes, a hallmark of secondary syphilis, are defined by a ring of scales encircling or contained within a circumscribed lesion. Classically, they present on the palms and soles. One is visible on our patient's right sole (indicated by the arrow). More may have been present but overtaken by excoriation. They were found in greater numbers over his trunk and extremities. [file 1124033.f1.zip › 1124033.f1/Figure3 (1).pdf]
